# Molecular targeted therapy for anticancer treatment

**DOI:** 10.1038/s12276-022-00864-3

**Published:** 2022-10-12

**Authors:** Hye-Young Min, Ho-Young Lee

**Affiliations:** College of Pharmacy and Research Institute of Pharmaceutical Sciences, Seoul National University, Seoul, Republic of Korea

**Keywords:** Targeted therapies, Targeted therapies

## Abstract

Since the initial clinical approval in the late 1990s and remarkable anticancer effects for certain types of cancer, molecular targeted therapy utilizing small molecule agents or therapeutic monoclonal antibodies acting as signal transduction inhibitors has served as a fundamental backbone in precision medicine for cancer treatment. These approaches are now used clinically as first-line therapy for various types of human cancers. Compared to conventional chemotherapy, targeted therapeutic agents have efficient anticancer effects with fewer side effects. However, the emergence of drug resistance is a major drawback of molecular targeted therapy, and several strategies have been attempted to improve therapeutic efficacy by overcoming such resistance. Herein, we summarize current knowledge regarding several targeted therapeutic agents, including classification, a brief biology of target kinases, mechanisms of action, examples of clinically used targeted therapy, and perspectives for future development.

## Cancer: A focused approach to therapy

Molecular targeted therapies have transformed cancer treatment, enabling personalized treatment of tumors in which growth is being powered by specific mutations. In contrast to traditional chemotherapies, which are toxic to both healthy and tumor cells, these targeted agents are designed to specifically block the effects of particular signaling proteins whose activity is largely restricted to cancerous tissue. Hye-Young Min and Ho-Young Lee at Seoul National University, South Korea, briefly review the history of the development of these drugs, and outline the mechanisms by which different targeted therapies work and how the acquisition of new tumor mutations can confer drug resistance. They also examine recent progress in developing agents that target proteins that have previously proven challenging therapeutic targets, such as the well-known oncogene *KRAS*, and explore potential opportunities for future therapeutic development.

## Introduction

Cancer is one of the main causes of disease-related death worldwide. According to Global Cancer Observatory (GLOBOCAN) estimates of cancer incidence and mortality, there were approximately 19.3 million new cancer cases and almost 10.0 million cancer deaths in 2020 globally^1^. The cancer-related burden (such as incidence and mortality) is expected to be 28.4 million cases in 2040, which is a 47% increase compared with that in 2020, largely due to increases in risk factors, such as aging, socioeconomic development, overweight status, and smoking^1,2^. Therefore, it is necessary to develop efficacious treatment strategies for patients with cancer.

Several therapeutic modalities, such as surgery, radiation therapy, and systemic anticancer therapy, have been applied clinically for cancer treatment, either alone, in combination, or sequentially, depending on the stage, resectability, biology, comorbidities, and patient’s overall functional performance^3,4^. Systemic anticancer therapy, involving a wide range of anticancer drugs for treatment, palliation, symptom alleviation, and quality of life improvement, includes cytotoxic chemotherapy, hormonal agents, targeted therapy, and antitumor immunotherapy^5,6^. Cytotoxic chemotherapy inhibits the survival of actively proliferating cells by disrupting the synthesis of DNA and RNA, blocking mitosis, and/or forming covalent bonds with DNA, RNA, and proteins^7^, and it has been extensively used in adjuvant or neoadjuvant therapy as well as in palliative therapy^7^. Due to the disadvantages of chemotherapy, including side effects and toxicity associated with nonselective action against actively proliferating normal cells^2,8^, there has been innovative development of ‘targeted’ cancer treatment with increased cancer cell specificity^8^. Targeted therapy may include the following: conventional molecular targeted agents, such as small molecule inhibitors or antibodies that specifically inhibit signal transduction pathways involved in growth, proliferation, and survival^9,10^; hormonal agents such as estrogen receptor (ER) antagonists and aromatase inhibitors, which have been used for treatment of hormone receptor (HR)-dependent breast cancer and male and female reproductive cancers^11^; immune checkpoint inhibitors [e.g., antibodies against programmed cell death protein 1 (PD-1), programmed death-ligand 1 (PD-L1), or cytotoxic T-lymphocyte-associated protein 4 (CTLA-4)], which activate host antitumor immunity in a direct or indirect manner^8,12^; and even targeted cytotoxic therapy that interferes with a specific cellular target (e.g., methotrexate, a dihydrofolate reductase inhibitor)^10^. Despite the anticancer effectiveness of these targeted therapies, these drugs are only applicable for patients harboring targetable driver mutations or aberrations^13,14^. In addition, side effects or toxicity caused by unexpected cross-reactivity with normal cells and emergence of intrinsic or acquired drug resistance hamper their effectiveness^13,14^. Notwithstanding some limitations, targeted therapy has resulted in remarkable survival benefits in some types of cancer and has led to a revolution in the fundamental concept of cancer treatment, providing the fundamental backbone for evolution toward precision or personalized medicine in cancer^13,15^. Herein, we summarize current knowledge with respect to molecular targeted therapy, including the history, types, and mechanism of action, and provide examples of clinically available targeted therapy. In this paper, ‘targeted therapy’ is confined to conventional molecular targeted therapy (signal transduction inhibitors).

### Brief history of molecular targeted therapy

**Fig. 1:**
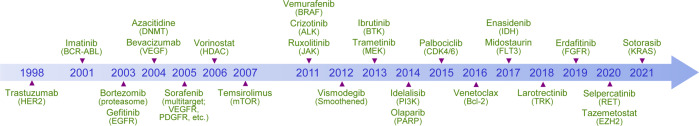
Timeline for the approval of selected molecular targeted therapeutic agents. The first FDA-approved targeted therapeutic agent for each cellular target (denoted in blankets) is indicated in the timeline.

Paul Ehrlich first proposed the concept of targeted therapy in the 1890s as a “magic bullet” that would be completely specific for the target and thus safe without any additional toxicity^14,16^. This theory was initially applied to infectious diseases but not to anticancer therapy due to insufficient knowledge of the etiology and biology of cancer^14,16^; however, this concept has since been expanded to cancer treatment^14,16^. Trastuzumab, an anti-HER2 monoclonal antibody, and imatinib, a small molecule tyrosine kinase inhibitor targeting the BCR-ABL fusion-mediated aberrantly activated ABL kinase, were developed and clinically approved in 1998 and 2001 for treatment of HER2-positive breast cancer and Philadelphia chromosome-positive chronic myelogenous leukemia, respectively^14,17–19^. The success of imatinib in the clinic has served as the paradigm for extensive use of small molecule kinase inhibitors as anticancer therapy^8,17^, and a number of anticancer molecular targeted therapies have been approved for clinical use in cancer patients^8,17^. The timeline for the development of the main molecular targeted therapy is illustrated in Fig. 1.

### Types, mechanisms of action and resistance, and adverse effects/toxicity of molecular targeted therapy

To date, numerous molecular targeted therapeutic agents have been used clinically for cancer treatment. The classification of molecular targeted therapeutic agents and their targets, mechanism of action, side effects, and toxicity are described below.

#### Types of molecular targeted therapy

**Fig. 2:**
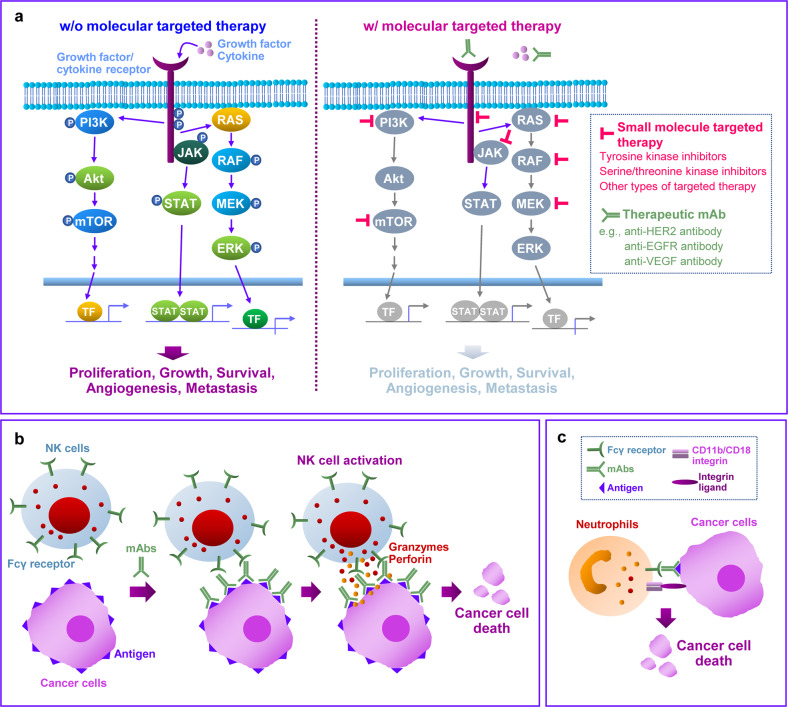
Mechanism of the anticancer effect of molecular targeted therapy. **a** Schematic diagrams of the main protumor signal transduction pathways and their inhibition by molecular targeted therapeutic agents. **b**, **c** Schematic diagrams for antibody-dependent cellular cytotoxicity **b** and trogoptosis **c**. See the text and relevant references for details.

The two major types of molecular targeted therapy are monoclonal antibodies (mAbs) and small molecule kinase inhibitors (SMKIs)^8,14^. mAbs target extracellular ligands (e.g., bevacizumab targets vascular endothelial growth factor [VEGF]), membrane receptors (e.g., trastuzumab targets HER2 and cetuximab; panitumumab targets EGFR), and membrane-bound proteins (e.g., rituximab targets CD20), acting through ligand-binding blockade, ligand‒receptor interaction neutralization, or target molecule internalization/degradation^14,20^. Except for inhibitors targeting nonkinase cellular proteins (e.g., mutated KRAS and proteasome) or epigenetic modulators (e.g., histone deacetylases), most SMKIs suppress protein kinases involved in the transformation, growth, proliferation, and survival of cancer cells. As deregulation of protein kinases (e.g., activation by gain-of-function genetic mutation, gene amplification, autonomous activation, and chromosomal rearrangement) has been associated with cancer development and progression^21–24^, protein kinases have been regarded as important targets for developing molecular targeted therapies. Protein kinases are classified into receptor tyrosine kinases, nonreceptor (cytoplasmic) tyrosine kinases, serine/threonine kinases, and lipid kinases based on their subcellular localization, substrate type, and hallmark roles in cancer^21^ (Fig. 2). A detailed explanation of the signal transduction by receptor tyrosine kinase is described in previous studies^24,25^.

SMKIs block the enzymatic activity of the aforementioned kinases via several modes of action^26^. Type I kinase inhibitors bind to the ATP-binding pocket of the active conformation of the enzyme [DFG (Asp-Phe-Gly)-in and αC-helix-in]^26^, whereas type I^1/2^ or type II inhibitors bind the enzyme in an inactive conformation (type I^1/2^: DFG-Asp in; type II: DFG-Asp out)^21,26^. Type III and type IV inhibitors allosterically suppress kinase activity by binding either to a site next to the ATP-binding pocket or one remote from the ATP-binding pocket located in the kinase substrate-binding site^21,26,27^. Type V inhibitors act as bivalent inhibitors binding to two different portions of the kinase lobe^21,26^. Type VI inhibitors covalently bind an enzyme to inhibit kinase activity^26,28^. A recent paper describes the detailed mode of action of each type of kinase inhibitor^26^, and some examples are listed in Table 1.

**Table 1:** Classes of selected kinase inhibitors^26^^,28^.

Class	Mechanism of action	Examples
Type I	Binding in the ATP-binding pocket of the active conformation of the enzyme (DFG-in and αC-helix-in)	cabozantinib, ceritinib, gefitinib, palbociclib, pazopanib, ponatinib, ruxolitinib, tofacitinib
Type I^1/2^ Type II	Binding in the ATP-binding pocket of the inactive conformation of the enzyme (type I^1/2^: DFG-Asp in; type II: DFG-Asp out)	dasatinib, imatinib, lapatinib, lenvatinib, nilotinib, regorafenib, sorafenib, sunitinib, vemurafenib
Type III Type IV	Allosteric inhibitors binding to a site in the kinase domain either next to the ATP-binding pocket or remote from the ATP-binding pocket	trametinib, everolimus, sirolimus, temsirolimus
Type V	Bivalent inhibitors that bind two different portions of the kinase lobe	lenvatinib^28^
Type VI	Covalent inhibitors	afatinib, ibrutinib

In Ref. ^26^, lenvatinib is classified as a type I^1/2^ inhibitor.

#### Mechanisms of the anticancer effects of molecular targeted therapy

Molecular targeted therapies achieve anticancer effects through various mechanisms, such as inhibition of cell proliferation, metastasis, and angiogenesis, induction of apoptosis, and reversal of multidrug resistance^2^ (Fig. 2a). Several molecular targeted therapeutic agents also facilitate host antitumor immunity by potentiating CD8^+^ T-cell recruitment and natural killer cell cytotoxicity, downregulating immunosuppressive myeloid cells, and inducing immunogenic cell death, either alone or in combination with chemotherapeutic agents^29^. Therapeutic mAbs create a bridge between tumor cells and immune cells *via* Fab region-mediated binding to a target protein of tumor cells and recognition of immune cells through the Fc region of antibodies^30^, resulting in opsonization and antibody-dependent cellular cytotoxicity (ADCC) toward tumor cells^30^ (Fig. 2b). A recent study demonstrated that neutrophils mediate trogoptosis (Fig. 2c), the phenomenon of transferring surface molecules of interacting cells onto immune cells^31,32^, which causes lytic/necrotic death of antibody-opsonized cancer cells^33^. mAbs and SMIs also exert immune cell-induced cytotoxic effects on cancer cells by activating complement and complement-dependent cytotoxicity^30,34^, facilitating antigen processing by increasing expression of major histocompatibility complex molecules^30,35,36^ and regulating cytokine/chemokine expression^30,37^.

#### Mechanisms underlying resistance to molecular targeted therapy

The emergence of drug resistance is a major hurdle of efficacious anticancer treatment. Primary (intrinsic) resistance is defined as a refractory status to initial therapy due to intrinsic cellular, genetic, and/or epigenetic alterations. Hyperactivation of compensatory signaling pathways [e.g., truncated HER2 expression (p95HER2) for resistance to anti-HER2 mAbs^38^; *KRAS* mutation or *MET* amplification for resistance to anti-EGFR therapy^38,39^], mutations in kinase domains (e.g., *EGFR* exon 20 insertion for resistance to anti-EGFR therapy^38^), isoform switching (e.g., BRAF/CRAF switching for resistance to anti-BRAF therapy^40^), and metabolic reprogramming^40^ during disease development are involved in primary resistance to molecular targeted therapy.

Human cancers often exhibit substantial intratumor heterogeneity, which is a main driver for emerging acquired therapy resistance as a result of expansion of rare preexisting refractory populations during treatment in initial responders^39,41,42^. Various molecular and cellular alterations [e.g., development of secondary mutations [EGFR T790M and C797S^38,43,44^, BCR-ABL T315I^44^, BRAF V600E^40,44^, Bruton’s tyrosine kinase (BTK) C418S^44^, anaplastic lymphoma kinase (ALK) G1202R, and ROS1 G2032R and D2033N^44^], alterations in noncoding RNAs^44^, activation of bypassing signaling pathways, including MET, HER2, type I insulin-like growth factor receptor (IGF-1R), and AXL^43,45^, mutations in BRAF, PTEN, PIK3CA, and MAP2K1^43,45^, interaction with stromal cells in the tumor microenvironment^43,46^, alterations in E3 ubiquitin ligases^47^, reactivation of developmental processes, such as the epithelial-mesenchymal transition (EMT), acquisition of cancer stem cell (CSC)-associated phenotypes, and transdifferentiation to small-cell lung cancer^43,48^] have also been shown to induce acquired therapy resistance. The mechanisms of resistance to each molecular targeted therapy are summarized in Tables 2–6.

**Table 2:** Receptor tyrosine kinase inhibitors that have been clinically used for cancer treatment.

Target	Generic name (Code name)	Brand name (Company)	First approved indication (Year)	Additional indication	Drug resistance mechanism (selected)	Side effects/toxicity (selected)	References
EGFR^1^	Gefitinib (ZD1839)	Iressa (AstraZeneca)	Advanced NSCLC^2^ after failure of both platinum-based and docetaxel chemotherapies (2003)	Metastatic NSCLC harboring EGFR mutations (first-line therapy, 2015)	EGFR T790M mutation MET amplification HER2 amplification Small-cell lung cancer transformation	Skin rash, nausea, diarrhea, transaminitis, ILD^3^-like disorders, hematuria	^8,52,63,195–197^
EGFR	Erlotinib (OSI-744)	Tarceva (Roche/Astellas)	Locally advanced or metastatic NSCLC after failure of prior chemotherapy regimen (2004)	Metastatic NSCLC harboring EGFR mutations (first-line therapy, 2013)	EGFR T790M mutation HGF overexpression MET amplification HER2 amplification Small-cell lung cancertransformation	Skin rash, diarrhea, ocular toxicity	^8,50,52,64,195–197^
EGFR	Afatinib (BIBW2992)	Gilotrif (Boehringer Ingelheim)	Metastatic NSCLC with kinase activating mutations (2013)	Advanced squamous cell carcinoma of the lung after treatment with platinum-based chemotherapy (2016)	EGFR T790M mutation MET amplification EGFR V843I mutation	Skin rash, diarrhea	^196,198,199^
EGFR	Dacomitinib (PF-00299804)	Vizimpro (Pfizer)	Metastatic NSCLC with kinase activating mutations (2018)		EGFR T790M/C797S mutation	Skin toxicity, dermatitis acneiform, paronychia, diarrhea	^67,200,201^
EGFR	Osimertinib (AZD9291)	Targrisso (AstraZeneca)	1st- or 2nd-generation EGFR-TKI-refractory NSCLC (2015)	Advanced NSCLC with mutated EGFR, regardless of T90M mutation (2018)	Loss of EGFR T790M mutation EGFR C797S mutation MET amplification Wild-type EGFR amplification	Skin rash, diarrhea, mucositis/stomatitis, paronychia, pneumonitis, cardiac failure	^8,79,195,202–204^
EGFR	Lazertinib^4^ (YH25448)	Leclaza (Yuhan/Janssen)	Advanced or metastatic NSCLC (2021)		Loss of EGFR T790M mutation EGFR activating mutation/amplification EGFR C797S mutation	Skin rash, itchiness, paresthesia, muscle spasm, headache, diarrhea, anorexia	^72^
EGFR	Cetuximab	Erbitux (ImClone)	Metastatic CRC^5^ (2004)	Head and neck squamous cell carcinoma (2006)	RAS/BRAF mutation EGFR S492R mutation MET amplification PTEN loss	Infusion reactions, acneform skin rash, nail disorder	^87–89,205–208^
EGFR	Panitumumab	Vectibix (Abgenix/Amgen)	Metastatic CRC (2006)		RAS/BRAF mutation MET amplification PTEN loss	Integument toxicity, skin toxicity, diarrhea	^88,89,206,208^
EGFR	Amivantamab (JNJ-61186372)	Rybrevant (Janssen Biotech)	Advanced NSCLC with EGFR exon 20 insertion mutations progressing after platinum-based chemotherapy (2021)			Infusion reactions ocular toxicity, peripheral edema, hypoalbuminemia	^79,80^
EGFR	Mobocertinib (TAK-788)	Exkivity (Takeda Pharmaceuticals)	Advanced NSCLC with EGFR exon 20 insertion mutations progressing after platinum-based chemotherapy (2021)			Diarrhea, skin toxicity	^79,81^
HER2	Trastuzumab	Herceptin (Genentech/Roche)	Metastatic breast cancer (1998)	Locally advanced unresectable or metastatic HER2^+^ gastric or gastroesophageal junction (GEJ) adenocarcinoma in combination with pembrolizumab (2021)	Truncation of HER2 extracellular domain (p95 HER2) PTEN loss IGF-1R expression PIK3CA mutation	Cardiotoxicity	^8,56,90,209^
HER2	Pertuzumab	Perjeta (Genentech/Roche)	HER2^+^ early breast cancer (EBC) with high risk of recurrence (2017)			Diarrhea, nausea, alopecia, fatigue, peripheral neuropathy, vomiting	^8,90,210^
HER2	Zanidatamab (ZW25)	(Zymeworks)	Advanced/metastatic HER2-expressing biliary tract cancers			Diarrhea, infusion-related reactions	^91^
HER2	Lapatinib (GW-572016)	Tykerb (GlaxoSmithKline/ Novartis)	HER2^+^ metastatic breast cancer progressing with prior therapy (in combination with capecitabine, 2007)	Triple-positive metastatic breast cancer (in combination with letrozole, 2010)	Crosstalk with ER HER2 mutation PIK3CA mutation AXL elevation HER2 L755S mutation	Diarrhea, skin rash, asymptomatic cardiotoxicity	^82,83,209,211^
HER2	Neratinib (HKI-272)	Nerlynx (Puma Biotechnology)	Extended adjuvant therapy for HER2^+^ breast cancer (2017)	Advanced or metastatic HER2^+^ breast cancer progressing with prior therapy (in combination with capecitabine, 2020)	TORC1 hyperactivation RAS upregulation	Diarrhea	^56,84,209,211,212^
HER2	Tucatinib (ONT-380)	Tukysa (Seattle Genetics)	Advanced or metastatic HER2^+^ breast cancer (in combination with trastuzumab and capecitabine, 2020)		HER2 L755S mutation	Diarrhea cardiotoxicity	^85,211^
ALK ROS1 MET	Crizotinib (PF-02341066)	Xalkori (Pfizer)	Locally advanced or metastatic ALK^+^ NSCLC (2011)	ROS1-positive NSCLC (2016)	ALK mutation (G1269A, C1156Y, E1210K, I1171T, S1206C/Y, I1151T/N/S, 1174 C/L/V, V1180L, L1196M)	Nausea, vomiting, diarrhea, visual disturbance, sinus bradycardia, liver enzyme abnormalities	^79,96,98,99,213^
ALK	Ceritinib (LDK378)	Zykadia (Novartis)	ALK^+^ metastatic NSCLC after failure of crizotinib therapy (2014)	ALK^+^ metastatic NSCLC (first-line therapy, 2017)	ALK mutation (G1202R, F1174C/L/V, 1151Tins, L1152P, C1156Y)	Diarrhea, nausea, vomiting, fatigue, elevated level of transaminase	^95,96,213^
ALK	Alectinib (CH5424802)	Alecensa (Chugai Pharmaceutical/ Roche	ALK-rearranged advanced/recurrent NSCLC with crizotinib resistance (2015)	ALK^+^metastatic NSCLC (first-line therapy, 2017)	ALK mutation (G1202R, V1180L and I1171T/N/S) MET amplification	Photosensitivity, dysgeusia, myalgia, upregulated creatinine phosphokinase	^79,96,213^
ALK EGFR	Brigatinib (AP26113)	Alunbrig (ARIAD Pharmaceuticals)	ALK-rearranged metastatic NSCLC (2017)		ALK double mutation (G1202R, E1210K and S1206C or D1203N)	Pneumonitis, nausea, diarrhea, fatigue	^79,101,102,213^
ALK ROS1	Lorlatinib (PF-6463922)	Lorbrena (Pfizer)	ALK-rearranged metastatic NSCLC (2018) (second/third-line treatment, accelerated approval)	ALK^+^ metastatic NSCLC (2021) (regular approval)	Compound ALK mutation including G1202R, I1171N/T/S, and L1198F ALK L1256F mutation MET amplification	Edema, cholesterolemia, peripheral neuropathy, hypertriglyceridemia, CNS effects	^8,92,203^.
MET	Capmatinib (INC280)	Tabrecta (Novartis)	Metastatic NSCLC harboring MET exon 14 skipping (2020)		MET mutation at D1228 and Y1230 (D1228 A/E/G/H/N/V/Y, Y1230 C/D/H/N/S)	Nausea, diarrhea, peripheral edema, hypoalbuminemia, increased blood creatinine	^79,99,214^
MET	Tepotinib (EMD 1214063)	Tepmetko (Merck)	Metastatic NSCLC harboring MET exon 14 skipping (2021)		MET mutation at D1228 and Y1230 (D1228 A/E/G/H/N/V/Y, Y1230 C/D/H/N/S)	Nausea, vomiting, peripheral edema, hypoalbuminemia, increased blood creatinine	^79,99,214^
TRK	Larotrectinib (LOXO-101)	Vitrakvi (Loxo Oncology/Bayer)	Locally advanced or metastatic solid tumors with NTRK gene fusion (2018)		TRKA F589L/G595R/G667C, TRKC G623R/G696A mutation	Upregulation of serum AST/ALT, dizziness, fatigue, nausea, constipation	^106,107^
TRK ALK ROS1	Entrectinib (RXDX-101)	Rozlytrek (Genentech)	Solid tumors with NTRK gene fusion and NSCLC harboring ROS1 rearrangement (2019)		TRKA G595R/G667C, TRKC G623R mutation	Fatigue, dysgeusia, nausea, vomiting, paresthesia, myalgia, diarrhea	^106,107^
FLT3 c-Kit PDGFR Src VEGFR	Midostaurin (PKC412, CGP 41251)	Rydapt (Novartis)	AML^6^ harboring FLT3 mutations (2017)		FLT3 N676K, F691L mutation FLT3 ligand overexpression RAS/MAPK mutation JAK, PI3K/Akt activation	Nausea, febrile neutropenia, mucositis, vomiting, headache, petechiae, fever	^109,215–217^
FLT3 AXL	Gilteritinib (ASP2215)	Xospata (Astellas Pharma)	FLT3-mutated refractory AML (2018)		FLT3 F691L mutation RAS/MAPK mutation JAK, PI3K/Akt activation	upregulation of hepatic transaminase/creatine phosphokinase, edema, cytopenia, febrile neutropenia	^109,215–217^
VEGFRs PDGFR-β c-Kit FLT3 RET RAFs	Sorafenib (BAY 43-9006)	Nexavar (Bayer/Onyx Pharmaceuticals)	Advanced RCC^7^ (2005)	HCC^8^ (2008) Locally recurrent or metastatic, progressive DTC^9^ refractory to radioactive iodine treatment (2013)	FLT3 F691L, Y842C/H, D835F/V/Y mutation FLT3 ligand overexpression JAK, PI3K/Akt activation	Hand-foot syndrome, asthenia, gastrointestinal irritation, cytopenia, infection, diarrhea, cardiovascular toxicity, fatigue	^109,215–217^
PDGFR-α/β VEGFR1/2/3 CSF-1R c-Kit, RET FLT3	Sunitinib (SU11248)	Sutent (Pfizer)	Advanced RCC (2006) Imatinib-resistant GIST^10^ (2006)	Pancreatic neuroendocrine tumor (2011)	Angiogenic factor upregulation Autophagy Metabolic adaptation Stromal cell recruitment	Mucositis, diarrhea, skin abnormality, taste alteration	^8,126,218,219^
VEGFR1/2/3 PDGFR-α/β FGFR1 FGFR3 c-Kit	Pazopanib (GW786034)	Votrient (GlaxoSmithKline/Novartis)	Advanced/metastatic RCC (2009)	Advanced soft-tissue sarcoma previously treated with chemotherapy (2012)	Angiogenic factor upregulation Stromal cell recruitment	Hepatic injury, fatigue, hand-food syndrome, myelosuppression	^220^
VEGFR1/2/3 PDGFR-α FGFRs c-Kit RET	Lenvatinib (E7080)	Lenvima (Eisai/Merck)	Progressive radioactive iodine-refractory thyroid cancer (2015)	Advanced RCC (recurrent or metastatic) (2016) Unresectable HCC (2018) Advanced RCC in combination with pembrolizumab (2021)	Angiogenic factor upregulation Stromal cell recruitment	Hypertension, diarrhea, fatigue/asthenia	^220^
MET VEGFR2 c-Kit RET AXL Tie2 FLT3	Cabozantinib (XL184)	Cometriq (capsule) Cabometyx (tablet) (Exelixis)	Cometriq: medullary thyroid cancer (2012) Cabometyx: RCC (2016)	Cabometyx: HCC (second-line, 2019)	Angiogenic factor upregulation Stromal cell recruitment	Diarrhea, palmar-plantar erythrodysesthesia syndrome	^220^
VEGFRs	Axitinib (AG 013736)	Inlyta (Pfizer)	Advanced or metastatic RCC (2012)		Angiogenic factor upregulation Stromal cell recruitment	Hypertension, diarrhea, fatigue	^220^
VEGFR2 EGFR3 GFR ET	Vandetanib ZD6474)	Zactima aprelsa AstraZeneca)	Medullary thyroid cancer (2011)		RET V804M/L mutation Activation of RAS/ RAF/MEK pathway	Diarrhea, skin rash, folliculitis, nausea, fatigue, hypertension, QT interval prolongation	^221^
VEGFR1/2/3 Tie2 PDGFR-α/β FGFR1/2 c-Kit RET RAFs	Regorafenib BAY 73-4506)	Stivarga (Bayer)	Metastatic CRC (2012)	Advanced GIST (2013) Advanced HCC (2018)	KIT V654A, D816V mutation	Hypertension, hand-food skin reaction, diarrhea, fatigue	^220,222^
VEGFR1/2/3 PDGFR-β c-Kit	Tivozanib (AV-951, KRN-951)	Fotivda (AVEO Pharmaceuticals /Kyowa Kirin)	Relapsed or refractory RCC (2021)		Infiltration of myeloid cells	Hypertension, hoarseness, fatigue, headache, diarrhea, rash	^223^
PDGFR-α c-Kit	Avapritinib (BLU-285)	Ayvakit (Blueprint Medicines)	Unresectable or metastatic GIST harboring PDGFRA exon 18 mutations, including D842V (2020)			Memory impairment, cognitive disorder, intracranial bleeding	^222^
PDGFR-α c-Kit	Ripretinib (DCC-2618)	Qinlock (Deciphera Pharmaceuticals)	Advanced GIST treated with three or more kinase inhibitors, including imatinib (2020)			Alopecia	^222^
FGFR	Erdafitinib (JNJ‑42756493)	Balversa (Janssen Pharmaceuticals)	Metastatic urothelial cancer (2018)	Metastatic or locally advanced bladder cancer with an FGFR3 or FGFR2 alteration (2019)	FGFR1 V561M/F mutation FGFR2 N549H mutation p.E565A and p.L617M single-nucleotide variants	Hyperphosphatemia, dry mouth, diarrhea, fatigue, stomatitis	^91,224,225^
FGFRs	Pemigatinib (INCB054828)	Pemazyre (Incyte Corporation)	Previously treated, unresectable, locally advanced, or metastatic cholangiocarcinoma with FGFR2 fusion or other rearrangements (2020)		FGFR1 V561M/F mutation FGFR2 N549H mutation	Hyperphosphatemia, dry mouth, diarrhea, fatigue, stomatitis	^91,224–226^
FGFRs	Futibatinib (TAS-120)	(Taiho Pharmaceutical)	Locally advanced/metastatic cholangiocarcinoma with FGFR2 gene rearrangement (2021)		p.E565A and p.L617M single-nucleotide variants FGFR2 V564F mutation	Hyperphosphatemia, dry mouth, diarrhea, paronychia,	^91,224,225^
FGFRs	Infigratinib (BGJ398)	Truseltriq (QED Therapeutics /Helsinn)	Locally advanced/metastatic cholangiocarcinoma with FGFR2 gene rearrangement (2021)		FGFR2 N549H, N550H/K, V564F, E565A, K660M, L618V, K641R mutation	Hyperphosphatemia, dry mouth, diarrhea, fatigue, stomatitis	^91,224,225^
FGFRs	Derazantinib (ARQ 087)	(Basilea Pharmaceutica /Merck)	Intrahepatic cholangiocarcinoma (2021)			Hyperphosphatemia, dry mouth, diarrhea, fatigue, stomatitis	^91,224,225^
RET	Selpercatinib (LOXO-292)	Retevmo (Eli Lilly/Loxo Oncology)	Metastatic RET fusion-positive NSCLC (2020) Advanced or metastatic thyroid cancer with RET alterations (2020)		RET mutation at G810, Y806	AST/ALT elevation, hypertension	^227,228^
RET	Pralsetinib (BLU-667)	Gavreto (Blueprint Medicines)	Metastatic RET fusion-positive NSCLC (2020)		RET mutation at G810, L730	AST/ALT elevation, anemia, hypertension	^227,228^

^1^EGFR: epidermal growth factor receptor.

^2^NSCLC: non-small cell lung cancer.

^3^ILD: interstitial lung disease.

^4^Approved in Republic of Korea.

^5^CRC: colorectal cancer.

^6^AML: acute myeloid leukemia.

^7^RCC: renal cell carcinoma.

^8^HCC: hepatocellular carcinoma.

^9^DTC: differentiated thyroid carcinoma.

^10^GIST: gastrointestinal stromal tumor.

**Table 3:** Monoclonal antibodies or recombinant proteins that inhibit angiogenesis modulators.

Class (Target)	Generic name (Code name)	Brand name (Company)	First approved indication (Year)	Additional indication (selected)	Drug resistance mechanism (selected)	Side effects/toxicity (selected)	References
Monoclonal antibody (VEGF)	Bevacizumab	Avastin (Genentech/Roche)	Metastatic CRC^1^ with standard chemotherapy treatment (2004)	Metastatic CRC with 5-FU-based therapy (second-line, 2006) Advanced nonsquamous NSCLC^2^ in combination with chemotherapy (2006) Metastatic RCC (2009) Recurrent GBM^3^ (2009) Metastatic cervical cancer (2014) Platinum-resistant recurrent ovarian cancer in combination with chemotherapy (2014)	Activation of the proangiogenic pathway Adaptation of an alternative mode of vessel formation	Bleeding, pulmonary hemorrhage, proteinuria, hypertension, would healing complications, cardiovascular toxicity, hypersensitivity	^229^
Monoclonal antibody (VEGFR2)	Ramucirumab (LY3009806, IMC-1121B)	Cyramza (Eli Lilly)	Advanced gastric cancer (2014) Aggressive NSCLC (2014)	Metastatic colorectal cancer in combination with FOLFIRI^5^ (2015) HCC (2019) EGFR mutated metastatic NSCLC (2020)		Neutropenia, thrombocytopenia, diarrhea, nausea, vomiting	^230^
Recombinant protein (VEGFs, VEGF-trap)	Aflibercept	Zaltrap, Eylea (Regeneron Pharmaceuticals)	Eylea: Wet age-related Macular Degeneration (2011) Zaltrap: previously treated metastatic CRC (2012)			Endophthalmitis, conjunctivitis, muscle volitantes, headache, arrythmia	^231^

^1^CRC: colorectal cancer.

^2^NSCLC: non-small cell lung cancer.

^3^GBM: glioblastoma multiforme.

^4^HCC: hepatocellular carcinoma.

^5^FOLFIRI: drug combination containing 5-fluorouracil, leucovorin calcium (folic acid), and irinotecan hydrochloride.

**Table 4:** Nonreceptor tyrosine kinase inhibitors that have been clinically used for cancer treatment.

Target	Generic name (Code name)	Brand name (Company)	First approved indication (Year)	Additional indication	Drug resistance mechanism (selected)	Side effects/toxicity (selected)	References
BCR-ABL PDGFR c-Kit	Imatinib (STI-571)	Gleevec (Novartis)	Ph^+1^ CML^2^ (2001)	GIST^3^ (2012) Ph^+^ ALL^4^ (2013)	BCR-ABL T315I mutation	Fatigue, rashes, fluid retention, bone pain, diarrhea	^8,135,137,138,145,232^
BCR-ABL c-Kit PDGFR SFKs^5^	Dasatinib (BMS-354825)	Sprycel (Bristol-Myers Squibb)	Ph^+^ ALL (2006)	Ph^+^ CML with resistance to or intolerance of prior therapy including imatinib (2009)	BCR-ABL T315I mutation	Neutropenia, thrombocytopenia, diarrhea, rash, fluid retention	^8,137,138,141,232,233^
BCR-ABL c-Kit PDGFR ARG DDR1 NQO2 EPHB4	Nilotinib (AMN107)	Tasigna (Novartis)	Ph^+^ CML with resistance or intolerance to existing therapies (2007)		BCR-ABL T315I mutation	Thrombocytopenia, myalgia, headache	^137–139,141^
BCR-ABL SFKs^5^ c-Kit PDGFR	Bosutinib (SKI-606)	Bosulif (Pfizer)	Ph^+^ CML with resistance or intolerance to imatinib (2012)		BCR-ABL T315I mutation	Diarrhea, nausea, vomiting	^135,137,138,140^
BCR-ABL PDGFR	Radotinib^6^ (IY-5511)	Supect (Ilyang Pharmaceutical)	CML (2012)		BCR-ABL T315I mutation	Thrombocytopenia, anemia, fatigue, asthenia, nausea, myalgia, pruritis	^8,142,234^
BCR-ABL FLT3 c-Kit VEGFR PDGFR Src	Ponatinib (AP24534)	Iclusig (ARIAD Pharmaceuticals)	Resistant or intolerant CML and Ph^+^ ALL (2012)		BCR-ABL compound mutation at T315, E255	Diarrhea, nausea, vomiting, headache	^135,137,145^
BCR-ABL	Asciminib (ABL001)	Scemblix (Novartis)	Ph^+^ CML (2021)		BCR-ABL mutation at A337, W464, P465, V468, I502	Diarrhea, nausea	^143,144,232^
BTK	Ibrutinib (PCI-32765)	Imbruvica (Pharmacyclics/AbbVie/Janssen)	MCL^7^ (2013)	CML (2014) Waldenström’s Macroglobulinemia (2015) CLL^8^ (first line) and SLL^9^ (2016) Relapsed/refractory MZL^10^ (2017)	BTK C481S, T474I/M mutation	Atrial fibrillation, bleeding, hypertension, diarrhea, nausea, vomiting	^151,152,235,236^
BTK	Acalabrutinib (ACP-196)	Calquence (Acerta Pharma/ AstraZeneca)	Relapsed/refractory MCL (2017)	Relapsed/refractory CLL (2019)	BTK C481S mutation	Atrial fibrillation, bleeding, hypertension, diarrhea, nausea, vomiting	^151,152,235,236^
BTK	Zanubrutinib (BGB-3111)	Brukinsa (BeiGene)	MCL (2019)	Waldenström’s Macroglobulinemia (2021) Relapsed/refractory MZL (2021)	BTK C481S mutation	Diarrhea, nausea, vomiting	^151,152,235,236^
JAK	Ruxolitinib (INC424)	Jakafi (Incyte/Novartis)	Myelofibrosis (2011)			Cytopenia, diarrhea, nausea, vomiting	^154,155,237^
JAK	Fedratinib (SAR302503, TG101348)	Inrebic (Celgene/Bristol-Myers Squibb)	Myelofibrosis (2019)			Diarrhea, nausea, vomiting	^154,155,237^

^1^Ph^+^: Philadelphia chromosome-positive.

^2^CML: chronic myeloid leukemia.

^3^GIST: gastrointestinal stromal tumor.

^4^ALL: acute lymphocytic leukemia.

^5^SFKs: Src-family kinases.

^6^Approved in Republic of Korea.

^7^MCL: mantle cell lymphoma.

^8^CLL: chronic lymphocytic leukemia.

^9^SLL: small lymphocytic lymphoma.

^10^MZL: marginal zone lymphoma.

**Table 5:** Serine/threonine kinase inhibitors that have been clinically used for cancer treatment.

Target	Generic name (Code name)	Brand name (Company)	First approved indication (Year)	Additional indication	Drug resistance mechanism (selected)	Side effects/toxicity (selected)	References
KRAS	Sotorasib (AMG 510)	Lumakras (Amgen)	Locally advanced or metastatic NSCLC harboring G12C-mutant KRAS with at least one prior systemic therapy (2021)		BRAF/RAS mutation KRAS G12V, G13D mutation	Nausea, vomiting, diarrhea, elevated aminotransferase level, fatigue, arthralgia	^163,164,238^
BRAF	Vemurafenib (PLX4032)	Zelboraf (Genentech)	Melanoma harboring V600E-mutant BRAF (2011)	Advanced melanoma with BRAF mutation in combination with cobimetinib (2015)	NRAS mutation CRAF overexpression secondary BRAF mutation MEK1/2 mutation	Rash, diarrhea, fatigue, arthralgia	^157,167,168,239–241^
BRAF	Dabrafenib (GSK2118436)	Tafinlar or Rafinlar (Novartis/GlaxoSmithKline)	Melanoma harboring V600E-mutant BRAF (2013)	Advanced melanoma with BRAF mutation in combination with trametinib (2014) BRAF V600E-mutant metastatic NSCLC^1^ in combination with trametinib (2017) BRAF V600E-mutant anaplastic thyroid cancer in combination with trametinib (2018)	NRAS mutation CRAF overexpression secondary BRAF mutation MEK1/2 mutation	Rash, diarrhea, fatigue, arthralgia	^157,167,168,239–241^
BRAF	Encorafenib (LGX818)	Braftovi (Novartis/Array BioPharma)	Unresectable or metastatic melanoma with BRAF mutations in combination with binimetinib (2018)	BRAF V600E-mutant metastatic CRC^2^ in combination with cetuximab (2020)	NRAS mutation CRAF overexpression secondary BRAF mutation MEK1/2 mutation	Rash, diarrhea, fatigue, arthralgia	^157,167,168,239–241^
MEK	Trametinib (GSK1120212, JTP-74057)	Mekinist (GlaxoSmithKline/Novartis)	BRAF V600E-mutant advanced melanoma (2013)	Advanced melanoma with BRAF mutation in combination with dabrafenib (2014) BRAF V600E-mutant metastatic NSCLC^1^ in combination with dabrafenib (2017) BRAF V600E-mutant anaplastic thyroid cancer in combination with dabrafenib (2018)	RTK reactivation PI3K, STAT3 activation	Rash, diarrhea, fatigue, arthralgia	^157,167,168,239–241^
MEK	Cobimetinib (GDC-0973, RG7420)	Cotellic (Genentech)	Advanced melanoma with BRAF mutation in combination with vemurafenib (2015)		RTK reactivation PI3K/STAT3 activation	Rash, diarrhea, fatigue, arthralgia	^157,167,168,239–242^
MEK	Binimetinib MEK162, ARRY-162, ARRY-438162)	Mektovi (Array Biopharma)	Unresectable or metastatic melanoma with BRAF mutations in combination with encorafenib (2018)		RTK reactivation PI3K/STAT3 activation	Rash, diarrhea, fatigue, arthralgia	^157,167,168,239–241^
MEK	Selumetinib (AZD6244, ARRY-142886)	Koselugo (Array Biopharma/ AstraZeneca)	Neurofibromatosis type 1 plexiform neurofibroma (2020)		RTK reactivation PI3K/STAT3 activation	Rash, diarrhea, fatigue, arthralgia	^157,167,168,239–241^
PI3Kδ	Idelalisib (CAL-101, GS-1101)	Zydelig (Gilead Sciences)	Relapsed CLL^3^ (2014)		RTK reactivation	Hyperglycemia, rash, stomatitis, fatigue, nausea, diarrhea	^171,172,243,244^
PI3Kγ PI3Kδ	Duvelisib (IPI-145, INK1197)	Copiktra (Intellikine/ Secura Bio)	Relapsed or refractory CLL, SLL^4^, and FL^5^ (2018)		Isoform switching Akt/mTOR activation Loss of PTEN	Hyperglycemia, rash, stomatitis, fatigue, nausea, diarrhea	^171,172,243,244^
pan-PI3K (p110α and p110δ)	Copanlisib (BAY 80–6946)	Aliqopa (Bayer)	Relapsed or refractory FL (2017)		RTK reactivation	Hyperglycemia, rash, stomatitis, fatigue, nausea, diarrhea	^171,172,243,244^
PI3Kα	Alpelisib (NVP-BYL719)	Piqray (Novartis)	HR^+^ and HER2^-^ advanced/metastatic breast cancer with a PIK3CA mutation with prior endocrine therapy (in combination with fulvestrant, 2019)		RTK reactivation	Hyperglycemia, rash, stomatitis, fatigue, nausea, diarrhea	^171,172,243,244^
mTOR	Sirolimus (AY-22989, rapamycin)	Rapamune (Pfizer)	Lymphangioleiomyomatosis (2015)	Locally advanced unresectable or metastatic malignant perivascular epithelioid cell tumor (2021)	RTK reactivation	Hyperglycemia, rash, stomatitis, fatigue, nausea, diarrhea	^171,172,243,244^
mTOR	Temsirolimus (CCI-779)	Torisel (Pfizer)	Advanced RCC^6^ (2007)		RTK reactivation	Hyperglycemia, rash, stomatitis, fatigue, nausea, diarrhea	^171,172,243,244^
mTOR	Everolimus (RAD001)	Afinitor (Novartis)	RCC after failure of sunitinib or sorafenib treatment (2009)	Advanced pancreatic neuroendocrine tumor (2011) HR^+^ and HER2^-^ breast cancer for use in combination with exemestane (2012) Subependymal giant-cell astrocytoma (2012)	RTK reactivation	Hyperglycemia, rash, stomatitis, fatigue, nausea, diarrhea	^171,172,243,244^
CDK4/6	Palbociclib (PD 0332991)	Ibrance (Pfizer)	Advanced or metastatic breast cancer (2015)	HR^+^ and HER2^-^ metastatic breast cancer (2016)	CDK4/6 overexpression	Fatigue, nausea, diarrhea, vomiting	^8,173,245^
CDK4/6	Ribociclib (LEE011)	Kisqali (Novartis)	HR^+^ and HER2^-^ metastatic breast cancer (2017)		CDK4/6 overexpression	Fatigue, nausea, diarrhea, vomiting	^8,174,245^
CDK4/6	Abemaciclib (LY2835219)	Verzenio (Eli Lilly)	HR^+^ and HER2^-^ advanced or metastatic breast cancer (2017)	HR^+^, HER2^-^, and node-positive early breast cancer with a high risk of recurrence and a Ki-67 score ≥20% (2021)	CDK4/6 overexpression	Fatigue, nausea, diarrhea, vomiting	^8,245,246^

^1^NSCLC: non-small cell lung cancer.

^2^CRC: colorectal cancer.

^3^CLL: chronic lymphocytic leukemia.

^4^SLL: small lymphocytic lymphoma.

^5^FL: follicular lymphoma.

^6^RCC: renal cell carcinoma.

**Table 6:** Additional targeted therapies that have been clinically used for cancer treatment.

Target	Generic name (Code name)	Brand name (Company)	First approved indication (Year)	Additional indication	Drug resistance mechanism (selected)	Side effects/toxicity (selected)	References
PARP	Olaparib (AZD2281)	Lynparza (AstraZeneca)	Advanced ovarian cancer (2014)	Maintenance treatment of ovarian cancer (2017, 2018, 2020) BRCA-mutated metastatic breast cancer (2018, 2022) Metastatic pancreatic cancer (2019, 2020)	Restoration of homologous recombination repair and ADP-ribosylation (PARylation) reversion mutations	Ileus, myelodysplastic syndrome, interstitial lung disease	^8,178,247^
PARP	Rucaparib (AG014699)	Rubraca (Clovis Oncology)	Advanced ovarian cancer (2016)	Maintenance treatment of ovarian cancer (2018)	Restoration of homologous recombination repair and ADP-ribosylation (PARylation) reversion mutations	Nausea, vomiting, diarrhea, constipation, red blood cell count decrease, photosensitivity, renal impairment, dysgeusia	^8,178,247^
PARP	Niraparib (MK-4827)	Zejula (Tesaro)	Recurrent ovarian cancer (2017)	Maintenance treatment for patients with platinum-responsive ovarian cancer regardless of biomarker status (2020)	Restoration of homologous recombination repair and ADP-ribosylation (PARylation) reversion mutations	Nausea, constipation, platelet/red blood cells count decrease, lymphangioleiomyomatosis	^8,178,247^
PARP	Talazoparib (BMN-673)	Talzenna (Pfizer)	BRCA1/2-mutated advanced or metastatic HER2^-^ breast cancer (2018)		Restoration of homologous recombination repair and ADP-ribosylation (PARylation) reversion mutations	Hematopoietic erythropenia, anemia, thrombocytopenia, pancytopenia, neutropenia	^8,178,247^
DNMT^1^	Azacitidine (5-azacytidine)	Vidaza (Pharmion Corporation)	MDS^2^ (2004)		Adaptive responses of the pyrimidine metabolism network	Fatigue, constipation, mucositis, pneumonia, febrile neutropenia	^8,247,248^
DNMT	Decitabine (NSC 127716)	Dacogen (Janssen-Cilag/Otsuka Pharmaceutical) Inqovi (oral tablet) (Otsuka Pharmaceutical)	Dacogen : MDS (2006)	Inqovi: MDS in combination with cedazuridine (2020)	Adaptive responses of the pyrimidine metabolism network	Fatigue, constipation, mucositis, pneumonia, febrile neutropenia	^8,247,249^
HDAC^3^	Vorinostat (SAHA)	Zolinza (Merck)	Relapse/refractory CTCL^4^ (2006)		Overexpression of Bcl-2 family proteins JAK/STAT3 pathways HDAC alterations Epigenetic alterations Protection of oxidative stress Alterations in apoptosis/autophagy	Diarrhea, fatigue, nausea, anorexia, dysgeusia, thrombocytopenia, pulmonary embolism, cardiac abnormalities	^250,251^
HDAC	Romidepsin (FK228, FR901228)	Istodax (Celgene Corp./Bristol-Myers Squibb)	CTCL (2009)	PTCL^5^ (2011)	P-glycoprotein-mediated drug efflux HDAC alterations Epigenetic alterations Protection of oxidative stress Alterations in apoptosis/autophagy	Thrombocytopenia, anemia, neutropenia, fatigue, nausea, vomiting, anorexia, tumor lysis syndrome	^250,251^
HDAC	Belinostat (PXD-101)	Beleodaq (Spectrum Pharmaceuticals)	PTCL (2014)		HDAC alterations Epigenetic alterations Alterations in apoptosis/autophagy	Nausea, vomiting, tumor lysis syndrome, hepatic failure, cardiac abnormalities	^250,251^
HDAC	Panobinostat (LBH-589)	Farydak (Novartis/Secura Bio)	MM^7^ (2015)		HDAC alterations Epigenetic alterations Protection of oxidative stress Alterations in apoptosis/autophagy	Severe diarrhea, nausea, vomiting, cardiac abnormalities	^250,251^
EZH2^6^	Tazemetostat (E7438/EPZ6438)	Tazverik (Epizyme)	Relapsed/refractory follicular lymphoma (2020)	Metastatic or locally advanced epithelioid sarcoma (2020)	EZH2 Y726F, C663Y mutation	Nausea, asthenia, fatigue, alopecia, dry skin, diarrhea, neutropenia, thrombocytopenia	^252^
IDH1^8^	Ivosidenib (AG-120)	Tibsovo (Servier Pharmaceuticals)	Relapse/refractory AML^9^ with an IDH1 mutation (2018)	Frontline in AML patients with comorbidities (2019) IDH1-mutated cholangiocarcinoma (2021)	Elevated 2-hydroxyglutarate Hypermethylation	QT interval prolongation, IDH differentiation syndrome, anemia, thrombocytopenia	^8,91,216,253^
IDH2	Enasidenib (AG-221)	Idhifa (Agios Pharmaceuticals)	Relapse/refractory AML with an IDH2 mutation (2017)		Elevated 2-hydroxyglutarate Hypermethylation	Hyperbilirubinemia, thrombocytopenia, IDH differentiation syndrome	^216,253^
Proteasome	Bortezomib (PS-341)	Velcade (Millennium/Takeda/Janssen Pharmaceutical)	Relapse/refractory MM (2003)		Proteasome mutation/overexpression Heat shock protein upregulation Autophagy Increased drug efflux Alterations in glutathione metabolism	Peripheral neuropathy, hematologic toxicities, diarrhea, fatigue, dyspnea, zoster reactivation	^254,255^
Proteasome	Carfilzomib (PR-171)	Kyprolis (Onyx Pharmaceuticals)	Advanced MM (2012)		Proteasome mutation Autophagy Increased drug efflux	Hematologic toxicities, pneumonia, hyponatremia, fatigue, hypophosphatemia, infusion reactions, chest pain, heart failure	^254,255^
Proteasome	Ixazomib (MLN2238)	Ninlaro (Takeda)	MM (2015)		Proteasome mutation Autophagy	Hematologic toxicities, fatigue, rash, decreased appetite, diarrhea, vomiting	^254,255^
Bcl-2	Venetoclax (ABT-199)	Venclexta (AbbVie/Genentech)	CLL^10^ (2016)	AML (2018)	BCL2 mutation Activation of the MAPK/Akt pathway Deregulation of energy metabolism Interaction with stromal cells	Bone marrow suppression, nausea, vomiting, diarrhea	^8,256^
Smoothened	Vismodegib (GDC-0449)	Erivedge (Genentech/Roche)	BCC^11^ (2012)		SMO mutations (e.g., D473H) SUFU/GLI2 copy number variation/mutation	Muscle spasm, weight loss, alopecia, dysgeusia	^8,257^
Smoothened	Sonidegib (NVP-LDE225)	Odomzo (Novartis)	Locally advanced BCC (2015)		SMO mutations SUFU/GLI2 copy number variation/mutation	Nausea, dysgeusia, anorexia, muscle spasm, fatigue, creatine kinase elevation	^8,257^
Smoothened	Glasdegib (PF-04449913)	Daurismo (Pfizer)	AML (2018)		SMO mutations SUFU/GLI2 copy number variation/mutation	Thrombocytopenia, anorexia, peripheral edema, fatigue, neutropenia	^8,257^

^1^DNMT: DNA methyltransferase.

^2^MDS: myelodysplastic syndrome.

^3^HDAC: histone deacetylase.

^4^CTCL: cutaneous T-cell lymphoma.

^5^PTCL: peripheral T-cell lymphoma.

^6^EZH2: enhancer of zeste homolog 2.

^7^MM: multiple myeloma.

^8^IDH: isocitrate dehydrogenase.

^9^AML: acute myeloid leukemia.

^10^CLL: chronic lymphocytic leukemia.

^11^BCC: basal cell carcinoma.

^12^ALCL: anaplastic large cell lymphoma.

#### Adverse effects and toxicity of molecular targeted therapy

Despite improved specificity for cancer cells, epidemiological studies have indicated that cancer patients who receive targeted therapy may experience various side effects and toxicity. The side effects of targeted therapy include asthenia, anorexia, dyspnea, diarrhea, nausea, vomiting, mucositis, skin rash, fever, hand-foot syndrome, fatigue, cardiotoxicity, hypertension, and bleeding^49,50^. Specifically, acneiform rash, a skin rash with an acne-like appearance, is a common side effect of anti-EGFR therapy^50,51^, and hypertension is a common side effect of bevacizumab and anti-VEGF receptor (VEGFR) therapy^52^. These common side effects are related to therapy response^52^. Severe toxicities, such as colitis, digestive perforation, toxic cardiomyopathy, pneumonitis/interstitial lung disease, acute respiratory distress syndrome, posterior reversible encephalopathy syndrome, necrotizing fasciitis, acute renal failure, and hypersensitivity, have been observed in patients receiving molecular targeted therapy, such as antiangiogenic agents, anti-EGFR therapy, and anti-HER2 therapy^53^. The side effects and toxicity of each molecular targeted therapy are summarized in Tables 2–6.

### SMKIs and mAbs in targeted cancer therapy

By focusing on U.S. Food and Drug Administration (FDA)-approved kinase inhibitors, target kinases and examples of clinically used inhibitors are briefly introduced below.

#### Receptor tyrosine kinase inhibitors

##### Inhibitors targeting the EGFR family

The human EGFR family comprises four members of the ErbB lineage of proteins (ErbB1/EGFR, ErbB2/HER2, ErbB3/HER3, and ErbB4/HER4)^8,54,55^. Except for HER2, due to its inability to bind ligand^54^, EGFR family members form homo- and heterodimers and are activated via binding of ligands, such as EGF, epiregulin, transforming growth factor-α (TGF-α), and neuregulins^8,54,55^. Approximately 25% of all types of breast cancer patients show HER2 gene amplification or overexpression^56^. EGFR kinase-activating mutations [e.g., exon 19 microdeletions and L858R point mutations in the cytoplasmic tyrosine kinase domain, truncation of extracellular domain (EGFRvIII)] as well as overexpression without genetic alterations may occur in solid tumors^57–59^. These genetic changes cause abnormal EGFR activation in a ligand-independent fashion^60^. Exon 19 microdeletions and L858R point mutations are commonly found in patients with non-small cell lung cancer (NSCLC), particularly in nonsmoking east Asian females^59,61^, and EGFRvIII is frequently observed in glioblastoma^57–59^. Additional EGFR mutations, including E884K, D761Y, T854A, and exon 20 insertion, have been detected in NSCLC and found to confer EGFR TKI resistance^62^.

Several EGFR TKIs have been developed over the past decades and are clinically used for treatment of patients with NSCLC harboring kinase-activating mutations (Table 2). Gefitinib and erlotinib are first-generation EGFR-TKIs^8,63,64^ that interact with the ATP-binding pocket of EGFR in either the active or inactive conformation^26^. Second-generation EGFR TKIs, such as afatinib and dacomitinib, are irreversible EGFR inhibitors that covalently bind to the ATP-binding pocket of EGFR^8,65,66^. Despite the great efficacy of first- and second-generation EGFR-TKIs in patients with kinase-activating mutations in EGFR^64,67^, the EGFR T790M mutation in exon 20^68^ is associated with acquired resistance to these first- and second-generation EGFR-TKIs^67,69^ (e.g., approximately half of NSCLC patients acquire resistance to first-generation EGFR-TKIs^69^). EGFR T790M provides advantages for the growth and survival of cancer cells^69^ and limits the therapeutic efficacy of EGFR TKIs through both steric hindrance and potentiated ATP binding^62,69^. Accordingly, EGFR TKIs targeting the T790M mutation have been developed and clinically utilized. Osimertinib, a third-generation EGFR TKI, inhibits EGFR kinase activity by forming a covalent bond with the cysteine-797 residue in the ATP-binding pocket and shows an approximately 200 times greater inhibitory effect on mutant EGFR [L858R or exon 19 deletion mutations additionally harboring T790M (L858R/T790M or exon19del/T790M)] than on wild-type EGFR^70,71^. Another third-generation EGFR TKI, lazertinib, is an orally available, CNS-penetrable, and irreversible EGFR TKI that inhibits EGFR T790M and kinase-activating mutations^72^. Despite the approval of these agents for clinical use, clinical trials evaluating recently developed EGFR TKIs, including canertinib (CI-1033, a pan-ErbB inhibitor), naquotinib (ASP8273, third-generation EGFR TKI), and rociletinib (CO-1686, third-generation EGFR TKI), have been discontinued owing to safety and risk/benefit issues^73^. Nonetheless, EGFR cysteine-797 mutation was found in 14% of NSCLC patients with acquired osimertinib resistance, leading to the development of fourth-generation EGFR TKIs^74,75^. Several fourth-generation EGFR TKIs (e.g., BLU-945, EAI045, and OBX02-011) that target EGFR T790M and EGFR C797S have been evaluated in preclinical and clinical settings^74,76–78^. Additionally, two inhibitors targeting EGFR exon 20 insertions, such as amivantamab and mobocertinib, have been recently approved for the treatment of patients with advanced NSCLC with progression after platinum-based chemotherapy^79–81^ (Table 2). SMKIs approved to date for clinical use in patients with HER2-positive breast cancer include lapatinib, neratinib, and tucatinib^8,56,82^. Lapatinib is an orally available TKI that reversibly interacts with the ATP-binding site of EGFR and HER2^83^, and neratinib is an orally available agent that covalently binds to the ATP-binding site of the tyrosine kinase domain of EGFR and HER2, resulting in irreversible EGFR/HER2 inhibition^84^. Tucatinib is an orally available, selective, and reversible HER2 inhibitor that competitively interacts with the ATP-binding site of HER2^85^. Several clinical trials for recently developed HER2-targeting TKIs are also ongoing^86^.

In addition to SMKIs, mAbs targeting EGFR and HER2 have been used in the clinic (Table 2). EGFR mAbs, including cetuximab and panitumumab, have been clinically used for treatment of patients with metastatic colorectal cancer^87–89^. HER2-targeting mAbs, such as trastuzumab and pertuzumab, are approved for clinical use in patients with HER2-positive breast cancer^90^. Recently, the HER2-bispecific antibody zanidatamab was approved for patients with HER2-expressing biliary tract cancers^91^, and several clinical trials for recently developed HER2-targeting monoclonal antibodies are ongoing^86^.

##### ALK inhibitors

ALK is an receptor tyrosine kinase (RTK) with structural homology to leukocyte tyrosine kinase (LTK), which belongs to the insulin receptor superfamily^92^. In normal tissues, ALK expression is predominant in the nervous system and is known to play an important role in physiological regulation of nervous system development and function^92,93^. Chromosomal rearrangement of the ALK gene and consequent generation of a fusion protein with a number of partner proteins, including echinoderm microtubule-associated protein-like 4 (EML4), nucleophosmin (NPM), tropomyosin 3 (TPM3), and tropomyosin 4 (TPM4), ALK gene amplification, or ALK mutations lead to overexpression of a constitutively activated ALK protein^92^. ALK alterations have been found in several types of cancer, such as anaplastic lymphoma, neuroblastoma, and NSCLC^92^. Approximately 3–7% of patients with NSCLC, especially for those with the adenocarcinoma subtype, have been reported to harbor ALK rearrangements; ALK mutations are mutually exclusive with KRAS and EGFR mutations^94,95^.

Several ALK inhibitors are currently available in the clinical setting (Table 2), and these drugs are approved for the treatment of NSCLC patients. Crizotinib, a first-generation ALK inhibitor, is an orally available ATP-competitive inhibitor that was clinically approved in 2011^95,96^. Crizotinib was initially developed as a MET inhibitor; however, based on the inhibitory effect of crizotinib on ALK at pharmacologically relevant concentrations and the structural homology of the ATP-binding site between ALK and ROS1, the clinical efficacy of crizotinib has been evaluated in patients carrying alterations in these genes^95,97^. Consequently, crizotinib has been used as a first- or second-line therapy in patients with NSCLC harboring ALK, ROS1, or MET alterations^96,98,99^. However, due to the rapid emergence of resistance to crizotinib and its weak ability to penetrate the central nervous system (CNS)^95,96,100^, additional ALK inhibitors have been developed. The second-generation ATP-competitive ALK/ROS1 inhibitor ceritinib and the ATP-competitive ALK inhibitor alectinib have been approved for treatment of patients with crizotinib resistance^96^. In contrast to crizotinib and ceritinib, alectinib can penetrate the CNS, curing NSCLC patients with brain metastasis and preventing progression of CNS metastasis^8,96^. Additional blood‒brain barrier (BBB)-permeable ATP-competitive ALK TKIs have been developed, including brigatinib, which is effective against FMS-like tyrosine kinase 3 (FLT3), insulin-like growth factor receptor (IGF-1R), EGFR, and several ALK mutations associated with resistance to crizotinib, ceritinib, and alectinib^101,102^, and lorlatinib, with inhibitory effects against all recognized ALK mutations except the L1198F mutation^8,92^.

##### MET inhibitors

MET is an RTK activated by hepatocyte growth factor (HGF) and mediates several physiological processes, such as embryogenesis and tissue repair; aberrant activation of MET by genetic alterations plays an important role in the proliferation, invasion, and metastasis of tumor cells^103^. Alterations in the MET gene, such as amplification, mutation, and alternative splicing (MET exon 14 skipping), have been detected in NSCLC and other solid tumors^8,99^. MET overexpression is associated with poor prognosis and resistance to chemotherapeutic agents, including EGFR targeted therapy^8,104^. In addition, MET gene exon 14 skipping leads to constitutive activation of the MET signaling pathway and confers sensitivity to MET inhibitors^105^. MET inhibitors, such as orally available ATP-competitive small-molecule TKIs and monoclonal antibodies, have been developed and evaluated in preclinical and clinical trials^99^. Among them, capmatinib and tepotinib are approved for clinical use in treatment of patients with metastatic NSCLC harboring MET exon 14 skipping^99^ (Table 2).

##### TRK and FLT3 inhibitors

Neurotrophic tyrosine receptor kinases (NTRKs) are oncogenes that encode tropomyosin receptor kinase (TRK) proteins, including TRKA, TRKB, and TRKC^106^. TRKs are activated by binding of intrinsic neurotrophin ligands, such as nerve growth factor (NGF) for TRKA, brain-derived neurotrophic factor (BDNF) and neurotrophin 4 (NT-4) for TRKB, and neurotrophin 3 (NT-3) for TRKC^106,107^. NTRK gene fusion caused by chromosomal rearrangements of NTRK genes with various fusion partners drives ligand-independent, constitutive activation of TRKs, which has been found in a wide range of cancer types, including mammary analog secretory carcinoma, secretory breast carcinoma, and infantile fibrosarcoma^106,107^. FLT3 (CD135), a class III RTK, is exclusively expressed in hematopoietic stem and progenitor cell populations^108^. Constitutive activation of FLT3 kinase through internal tandem duplications (FLT3-ITD) or missense mutations in the FLT3 tyrosine kinase domain^109^ has been observed in approximately 30% of patients with acute myeloid leukemia (AML) and a normal karyotype^109,110^. Several TKIs targeting TRKs (e.g., larotrectinib and entrectinib) or FLT3-ITD (e.g., midostaurin, sorafenib, and gilteritinib) have been developed and approved for clinical use. Examples are listed in Table 2.

##### Inhibitors targeting PDGFR, VEGFR, or FGFR family receptors and Ret

Tumor angiogenesis is a hallmark of cancer. Several growth factors and their receptors, such as platelet-derived growth factor (PDGF)/PDGFR, vascular endothelial growth factor (VEGF)/VEGFR, fibroblast growth factor (FGF)/FGFR, stem cell factor (SCF)/c-Kit, glial cell line-derived neurotrophic factor (GDNF)-family ligands/rearranged during transfection (RET), and angiopoietin/Tie^22,111^, regulate the growth, differentiation and migration of cancer cells and angiogenic activities of vascular endothelial cells^22,111^. PDGFs are members of the ‘cysteine knot’ growth factor superfamily, the members of which contain at least three disulfide bridges and forms homo- or heterodimers^112^. Five types of PDGF dimers (PDGF-AA, PDGF-AB, PDGF-BB, PDGF-CC, and PDGF-DD) have been identified, and these PDGFs transduce signals by binding to two isotypes of PDGFRs (PDGFR-α and PDGFR-β)^113^. PDGF-AA and PDGF-CC, ligands that bind to these PDGFRs with different affinities, have a high affinity for PDGFR-α, whereas PDGF-BB and PDGF-DD transduce signaling through PDGFR-β^113^. PDGFR-α plays both general and specific roles in the development of mesenchymal and fibroblastic cell compartments; PDGFR-β plays an important role in the formation of vascular mural cells, including vascular smooth muscle cells and pericytes^113^. Alterations in PDGFR-α and PDGFR-β are associated with vascular diseases and mesenchymal cell/fibroblast-driven pathological conditions, respectively^113^. Alterations in PDGFR-α, such as point mutations and amplification, exist in approximately 5% of patients with gastrointestinal stromal tumors (GISTs) and 5–10% of patients with glioblastoma multiforme^114^.

The VEGF family is composed of five glycoproteins, including VEGFA (VEGF), VEGFB, VEGFC, VEGFD (c-Fos-induced growth factor, FIGF), and placental growth factor (PIGF or PGF)^115^. VEGF is expressed as multiple alternative splicing isoforms, with pro- or antiangiogenic effects; among them, VEGF_165_ is the predominant proangiogenic isoform that is overexpressed in various solid tumors^115,116^. VEGF activates signal transduction by binding to VEGFR family receptors, VEGFR1 (FLT1), VEGFR2 (KDR), and VEGFR3 (FLT4)^25,115^. VEGFR2 is primarily expressed in vascular endothelial cells, and VEGF/VEGFR2 signaling plays a crucial role in angiogenesis by controlling vascular permeability, proliferation, migration, and survival of vascular endothelial cells^115,117^. VEGF also stimulates vasculogenesis in tumors by recruiting bone marrow-derived hematopoietic progenitor cells and endothelial progenitor cells^115^. VEGFC and VEGFD bind VEGFR3 and regulate lymphangiogenesis, contributing to metastatic spread through the lymphatic system^118^. In addition to these angiogenic effects on vascular endothelial cells, VEGF exerts several tumor-promoting effects, such as increased cancer cell proliferation, migration, invasion, stemness^119–121^, immune suppression^115^, and premetastatic niche formation^122^.

The FGF family growth factors, comprising 18 members that are categorized into six subfamilies, activate signal transduction by binding to FGFRs^123^. Five FGFRs (FGFR1-FGFR5) are known^123,124^. FGFR1-FGFR4 possess tyrosine kinase activity; in contrast, FGFR5 lacks the intracellular tyrosine kinase domain but acts as a coreceptor of FGFR1 and modulates ligand-mediated signaling^123–125^. Heparan sulfate glycosaminoglycan (HSGAG) binds to both FGF and FGFR, protecting FGFs from degradation, stabilizing the interaction between ligand and receptor, and facilitating dimerization of FGF-bound FGFR^123^. In cancer cells, aberrant activation of FGF/FGFR signaling caused by FGFR amplification, activating FGFR mutations, FGFR single-nucleotide polymorphisms, FGFR fusion protein formation with various binding partners, and deregulation of phospholipase Cγ1 (PLCγ1, FRS1) and FGFR substrate 2 (FRS2) all promote cell survival, cell proliferation, angiogenesis, acquisition of an epithelial-mesenchymal transition (EMT) phenotype, invasion, and metastasis in cancer cells^124,126^.

Similar to the aforementioned RTKs, ligand binding causes receptor dimerization, autophosphorylation, and activation of colony-stimulating Factor 1 receptor (CSF-1R)/FMS, c-Kit/stem cell factor receptor (SCFR), RET, and Tie^127–130^. Binding of GDNF family ligands to coreceptor GDNF family receptors (GFRα 1–4) is required to stimulate RET kinase^128^. In cancer, these signaling pathways promote the proliferation, survival, migration, and invasion of cancer cells and angiogenesis^127–130^. Alterations in CSF-1R, c-Kit, RET, and Tie caused by overexpression, genetic mutations, gene rearrangement, and fusion protein formation have been found in various types of cancer, including clear cell renal cell carcinoma (RCC, CSF-1R), GIST (c-Kit), acute myeloid leukemia (c-Kit), thyroid cancer (RET), and breast cancer (Tie1)^114,127,128,131,132^.

The kinase domain of RET is similar to that of VEGFR2, and PDGFR-α/β, c-Kit, CSF-1R, VEGFR1/2/3, Flt3, Tek, and Tie protein kinases are regulated by a similar autoinhibitory brake mechanism^133^; multikinase inhibitors concurrently targeting these kinases have been developed and clinically utilized. Examples are sorafenib, sunitinib, pazopanib, lenvatinib, regorafenib, vandetanib, cabozantinib, axitinib, tivozanib, avapritinib, ripretinib, erdafitinib, pemigatinib, infigratinib, derazantinib, futibatinib, selpercatinib, and pralsetinib. Moreover, monoclonal antibodies (e.g., bevacizumab and ramucirumab) or recombinant proteins (e.g., aflibercept) have been used clinically^134^. Several clinically approved inhibitors targeting these RTKs and additional angiogenesis inhibitors are listed in Tables 2 and 3.

#### Nonreceptor tyrosine kinase inhibitors

##### BCR-ABL and SFK inhibitors

Abelson (ABL) family kinases (ABL1 and ABL2) are nonreceptor tyrosine kinases that commonly contain a specific domain cassette consisting of the Src homology 3 (SH3) domain (a protein module that binds to proline-rich sequences), the SH2 domain (a protein module that binds to tyrosine phosphorylated sites), the tyrosine kinase domain (SH1 domain), the PXXP motif mediating interaction with SH3 domain-containing proteins, and the C-terminal F-actin binding domain^135,136^. ABL1, but not ABL2, additionally includes a DNA-binding domain, nuclear localization signal motifs, and nuclear export signal motif and mediates DNA damage repair^135,136^. ABL2 is mainly found at actin-rich sites, including focal adhesion and invadopodia in the cytoplasm, through its F-actin and microtubule-binding domains and mediates cytoskeletal remodeling^135,136^. Activation of ABL kinases is tightly regulated through autoinhibitory intramolecular interactions, intermolecular interactions with other proteins to disrupt or maintain autoinhibitory conformation, and posttranslational modifications such as trans- or Src-mediated tyrosine phosphorylation (e.g., activation of ABL1 by phosphorylation at Y245 and Y412), serine/threonine phosphorylation, acetylation, myristoylation, and polyubiquitination^135,136^. Oncogenic alterations in ABLs, including fusion protein formation caused by chromosome translocations in leukemia [e.g., BCR-ABL1 in Philadelphia chromosome-positive (Ph^+^) chronic myeloid leukemia (CML)] and amplification and somatic mutations in solid tumors, constitutively activate ABL-mediated signaling pathways and promote survival, proliferation, dedifferentiation, migration, and invasion in cancer cells^135^.

Several kinase inhibitors targeting the BCR-ABL fusion protein have been developed and used clinically (Table 4). Imatinib is an orally active first-generation BCR-ABL inhibitor. Imatinib is an ATP-competitive type II TKI that binds to the inactive conformation of the ABL kinase (DFG-out conformation^137^)^135,137^. Mutation in the ATP-interacting gatekeeper residue of the ATP-binding pocket (T315I) leads to maintenance of the active conformation of ABL and resistance to imatinib and related TKIs^137^. The amide substitution in the central aminophenyl ring of imatinib is crucial for tyrosine kinase inhibition, and the 6-methyl residue in the aminophenyl ring increases selectivity for BCR-ABL^137^. Due to the structural similarity among ABL, c-Kit, and PDGFRs (class III RTK)^25^, imatinib also inhibits PDGFR and c-Kit^8,135,137,138^. Second-generation BCR-ABL inhibitors have been developed and clinically utilized to overcome imatinib resistance caused by ABL kinase point mutations. Nilotinib is an ATP-competitive and orally active type II kinase inhibitor with greatly improved potency compared to imatinib^137,138^. Similar to imatinib, nilotinib inhibits the inactivated conformation of the ABL kinase, and resistance in the presence of BCR-ABL harboring the T315I mutation has been reported; however, nilotinib suppresses most imatinib-resistant BCR-ABL mutants and is not a substrate of drug influx/efflux transporters^137–139^. In addition, nilotinib displays inhibitory effects regarding activation of multiple kinases, such as c-Kit, PDGFR, the ABL-related kinase ARG, DDR1 kinase, oxidoreductase NQO2, and ephrin receptor EPHB4^138^. Bosutinib is an orally active and ATP-competitive dual SFK/ABL inhibitor^135,137,138^ showing similar inhibitory effects against mutated or amplified BCR-ABL associated with imatinib resistance^137,140^ and BCR-ABL harboring the T315I mutation^137,140^. Accordingly, bosutinib has been used for treatment of patients with Ph^+^ CML who are resistant to or intolerant of imatinib^141^. Other agents approved in the clinic include radotinib, an orally active second-generation BCR-ABL inhibitor that exhibits inhibitory effects on wild-type and some imatinib-resistant mutant forms of BCR-ABL and PDGFR^8,142^, and asciminib, an allosteric inhibitor that binds to the myristate pocket of BCR-ABL and is effective against T315I-mutant BCR-ABL^143,144^. Ponatinib, an orally available third-generation inhibitor against both wild-type and T315I-mutant BCR-ABL^135,137,145^, also displays inhibitory effects on the activity of multiple kinases, including FLT3, c-Kit, VEGFR, PDGFR, and Src^145^. Since 2012, ponatinib has been used for treatment of patients with T315I-positive CML (including accelerated phase, chronic phase, or blast phase) or those with T315I-positive Ph^+^ ALL^145^. Additional BCR-ABL-targeting inhibitors have been developed and evaluated preclinically and clinically^137^.

Src-family kinases (SFK: Blk, Fgr, Frk, Fyn, Hck, Lck, Lyn, Src, Yes, and Yrk) contain a conserved domain organization consisting of a myristoylated N-terminal segment (SH4 domain), followed by SH3, SH2, linker, and tyrosine kinase domains and a short C-terminal tail^146,147^. Among SFKs, Src, Fyn, and Yes are ubiquitously expressed; Hck, Lck, Lyn, Blk, Yrk, and Fgr are primarily expressed in hematopoietic cells and Frk-related kinases in epithelial-derived tissues. Similar to ABL, SFKs adopt an inactive conformation through autoinhibitory intramolecular interactions involving phosphorylation at Y527/Y530^146^. Dephosphorylation of Y527/Y530 causes destabilization of intramolecular interactions, leading to SFK activation by interaction with RTKs, G protein-coupled receptors, and focal adhesion kinase via its SH2 or SH3 domains and subsequent autophosphorylation at Y416/Y419^146,147^. Activated SFKs play a crucial role in cell proliferation, adhesion, migration, invasion, metastasis, angiogenesis, and therapeutic resistance in cancer and act as key nodes of multiple oncogenic signal transduction pathways^147,148^, indicating the potential of SFK targeting for efficacious anticancer therapeutic regimens. ABL in the active conformation is structurally similar to SFKs^138,149^, and dasatinib, which preferentially interacts with the active conformation of the ABL kinase domain^135,137,138^, shows inhibitory effects on SFKs^149^. Dasatinib targets multiple kinases, including c-Kit, PDGFR, and SFK (Src, Fgr, Fyn, Hck, Lck, Lyn, and Yes)^138,146,149^, but not BCR-ABL harboring the T315I mutation^137^. Currently, there are no clinically approved anticancer regimens targeting SFKs, and some clinical trials evaluating the effectiveness of SFK inhibitors are still ongoing^150^.

##### BTK and JAK inhibitors

BTK is a nonreceptor tyrosine kinase that plays an essential role in the development and function of B cells^151^. BTK contains five typical domains, including from the N-terminus to the C-terminus the pleckstrin homology (PH) domain required for binding to phosphatidylinositol lipids, the proline-rich TEC homology (TH) domain, a zinc-finger motif for optimal activity and stability of the protein, SH3 and SH2 domains, and the catalytic domain^151,152^. Antigen engagement by the B-cell receptor causes activation of BTK through phosphorylation at Y551 in the kinase domain by spleen tyrosine kinase (Syk), Lyn, or Src^152^, which leads to subsequent autophosphorylation at Y223 in the SH3 domain and activation of downstream signaling pathways, including phospholipase Cγ, mitogen-activated protein kinase (MAPK), nuclear factor kappa-light-chain-enhancer of activated B cells (NF-кB), and Akt, leading to regulation of B cell survival, proliferation, differentiation, and antibody secretion^151,152^ Overexpression and hyperactivation of BTK have been observed in a number of non-Hodgkin B-cell malignancies, including chronic lymphocytic leukemia (CLL), small lymphocytic lymphoma (SLL), and mantle cell lymphoma (MCL)^151,152^. The Janus kinase (JAK) family comprises the nonreceptor tyrosine kinases JAK1, JAK2, JAK3, and TYK2^153,154^. Cytokine binding to receptors leads to receptor dimerization and recruitment, trans-autophosphorylation, and activation of JAK, resulting in phosphorylation and activation of downstream signaling cascades such as phosphatidylinositol-3-kinase (PI3K)/Akt, MAPK, and signal transducer and activator of transcription (STAT) transcription factors^153–155^. Deregulation of JAK through hyperactivation and activating mutations (e.g., JAK2 V617F) has been reported in myeloproliferative neoplasms, including myelofibrosis^154^. Examples of clinically approved inhibitors targeting BTK (e.g., ibrutinib, acalabrutinib, and zanubrutinib) or JAK (e.g., ruxolitinib and fedratinib) are listed in Table 4.

#### Inhibitors targeting downstream signaling pathways: RAS inhibitor and serine/threonine kinase inhibitors

Activated tyrosine kinases trigger phosphorylation and activation of downstream signaling mediators that are mostly serine/threonine kinases. The main relevant downstream signaling pathways are the PI3K/Akt/mammalian target of rapamycin (mTOR) and RAS/RAF/MEK/ERK pathways. Alterations in several components of these pathways (e.g., RAS, RAF, MEK, and PI3K) have been found in various types of cancer and thus considered druggable targets^156–158^. Cyclins are also downstream effector molecules of these signaling cascades and play an important role in regulating cell cycle progression and various cellular processes, such as gene transcription, DNA damage repair, and metabolism, by associating with cyclin-dependent kinases (CDKs)^159^. Alterations in cyclins and CDKs have been observed in various cancer types, and several CDK inhibitors have been developed and approved for clinical use^160^. Examples of these targeted therapeutic drugs are described below.

##### RAS/RAF/MEK inhibitors

RAS is a guanine nucleotide-binding protein that plays an important role in cell proliferation and differentiation, and farnesylation of RAS by RAS farnesyltransferase (FTase) is crucial for RAS to associate with membranes and its transforming activity^161^. Mutations in RAS result in constitutive activation^161^. Among the three RAS isoforms (KRAS, HRAS, and NRAS), KRAS is the most frequently mutated isoform, and five mutations (G12D, G12V, G12C, G13D, and Q61R) are the most prominent RAS mutations observed in cancer patients^156^. Based on the important role of RAS FTase in the regulation of RAS transforming activity, several FTase inhibitors have been developed and evaluated, yet none of them have been clinically used because of limited efficacy^162^. Recently, a small molecule inhibitor targeting mutated KRAS (KRAS^G12C^) was developed and approved for clinical use. Sotorasib is an orally available inhibitor that binds to inactive guanosine diphosphate (GDP)-bound KRAS via a covalent bond between the C12 residue and the acrylamide warhead and noncovalent bonds between the isopropylpyridine substituent and a cryptic pocket comprising H95, Y96, and Q99 residues; this results in inhibition of KRAS^G12C^ without affecting wild-type KRAS^163,164^. Another KRAS^G12C^ inhibitor, adagrasib (MRTX849), is under clinical trial evaluation^165^.

Activated RAS in the GTP-bound state leads to association of RAF proteins, causing formation of RAF homo- or heterodimers, RAF phosphorylation, and consequent activation of the downstream signaling mediators MEKs and ERKs^157,166^. Among the three isoforms of RAF (ARAF, BRAF, and CRAF), mutations in BRAF, especially at the V600 residue (e.g., V600E) in the activation loop, are frequently observed in several types of cancer, including melanoma, papillary thyroid cancer, and colorectal cancer^157,166,167^. Indeed, the V600E mutation, which causes RAS-independent activation of BRAF, accounts for more than 90% of BRAF mutation cases in cancer^157,166,167^. Thus far, three RAF inhibitors and three MEK inhibitors have been used for anticancer treatment. Currently available RAF inhibitors target monomeric V600E-mutant BRAF; thus, for dimeric RAF, inhibition of one protomer by the drug paradoxically leads to transactivation of the other protomer and downstream signaling^157^. Therefore, a combination of MEK inhibitors (e.g., vemurafenib plus cobimetinib, dabrafenib plus trametinib, and encorafenib plus binimetinib) has been clinically utilized^168^. Examples of clinically approved BRAF and MEK inhibitors are listed in Table 5.

##### PI3K/mTOR inhibitors

The PI3K/Akt/mTOR pathway plays a central role in regulating cell proliferation, survival, growth, and metabolism^158,169^. Deregulation of the PI3K/Akt/mTOR pathway through mutation or amplification of PIK3CA (encoding the p110α subunit of PI3K), loss or inactivation of phosphatase and tensin homolog (PTEN), and hyperactivation of mTOR have been commonly found in various cancer types^158,169^ and related anticancer drug resistance^158,170^. Hence, inhibitors targeting PI3K, Akt, and mTOR have been evaluated in preclinical studies and clinical trials, and some inhibitors have been used clinically for cancer treatment.

Because of the specific expression of PI3K, p110γ, and p110δ subunits in the hematopoietic system, the association of the PI3K pathway with regulating B-cell receptor (BCR) signaling, and the undesirable toxicity of pan-PI3K or dual PI3K/mTOR inhibitors^171,172^, PI3K inhibitors that specifically target PI3Kδ or PI3Kγ have been employed for treatment of patients with lymphoma. Some mTOR inhibitors, especially rapamycin analogs (rapalogs) that form a complex with FK506-binding protein 12 (FKBP12) and inhibit mTORC1 (but not mTORC2) activity, have been approved for clinical use^8^. Additionally, ATP-competitive mTOR inhibitors have been developed and are under preclinical and clinical evaluation^8^. Examples of clinically utilized PI3K (e.g., idelalisib, duvelisib, copanlisib and alpelisib) and mTOR inhibitors (e.g., sirolimus, temsirolimus, and everolimus) are listed in Table 5.

##### CDK inhibitors

Among more than 20 members of CDK family proteins^159^, CDK4 and CDK6 (in complex with cyclin D) play a crucial role in promoting cell cycle progression by sequestering CDK inhibitors and inducing various proteins involved in cell cycle progression from G1 to S phase, DNA replication, chromatin structure, chromosome segregation, and the spindle assembly checkpoint through phosphorylation of various targets, including retinoblastoma protein (RB), and activating E2F-mediated transcription^160^. Hence, CDK4/6 has been considered attractive] for targeted anticancer therapy. Three CDK4/6 inhibitors have been used clinically for treatment of patients with HR-positive advanced breast cancer (Table 5). Palbociclib, ribociclib, and abemaciclib are orally available, reversible, and selective CDK4/6 inhibitors that have been used clinically in combination with an aromatase inhibitor for treatment of postmenopausal women with ER-positive and HER2-negative advanced or metastatic breast cancer^8,173,174^.

#### Other targeted anticancer agents

In addition to PARP inhibitors, other types of clinically used or recently approved targeted therapies, including epigenetic modulators (e.g., DNA methyltransferase inhibitors, histone deacetylase inhibitors, EZH2 inhibitors, and isocitrate dehydrogenase inhibitors), proteasome inhibitors, Bcl-2 inhibitors, and smoothened inhibitors, are summarized in Table 6.

##### PARP inhibitors

The PARP family plays a crucial role in regulating DNA repair processes upon the DNA damage response (DDR) and chromatin modulation^175,176^. PARP family proteins, especially PARP1 and PARP2, bind to DNA lesions and mediate poly-ADP ribosylation (PARylation) of chromatin and DNA damage response components, resulting in DNA repair by recruiting DNA repair effectors such as XRCC1^175,176^. After autoPARylation, PARP dissociates from DNA, and the DNA repair process is completed by recruitment of DNA repair proteins^176^. BReast CAncer gene 1 (BRCA1) and BRCA2 (BRCA1/2) are tumor-suppressor genes that play a key role in repair of double-strand DNA breaks *via* the conservative homologous recombination repair (HRR) process^175,177^. Mutations in BRCA1/2 genes have been found in some cancer types, including breast, ovarian, pancreatic, and prostate cancers^177^. Defects in BRCA function due to BRCA1/2 gene mutations cause loss of the HRR process and mediate the DNA repair process in a nonconservative manner, such as nonhomologous end joining, leading to DNA alteration^175^. As BRCA mutant cancer cells are vulnerable to blockade of the DNA repair process, treatment of BRCA-deficient cells with PARP inhibitors leads to unsustainable genomic instability and cancer cell death^176^. This synthetic lethal interaction between PARP blockade and BRCA1/2 mutation suggests a therapeutic strategy targeting PARP for treatment of cancer types harboring BRCA mutations. Based on these findings, some orally available PARP inhibitors, such as olaparib, rucaparib, niraparib, and talazoparib, have been clinically used for treatment of BRCA-mutated cancers, including ovarian, breast, and prostate cancers (Table 6)^8,178^. Additional investigations to evaluate the effectiveness of combinatorial treatment with chemotherapeutic agents, PI3K inhibitors, and anticancer immunotherapy have been conducted in preclinical and clinical settings^178^.

### Summary and future perspectives in the development of molecular targeted therapy

Owing to advances in molecular diagnosis, genome-wide analysis, and in-depth understanding of cancer biology, numerous tyrosine kinase inhibitors have recently been developed, tested preclinically and clinically, and utilized for cancer treatment in the clinic. Nevertheless, poor efficacy, toxicity, and tumor relapse due to drug resistance are major obstacles for targeted therapy-based efficacious anticancer treatment. Therefore, further investigation is required to develop efficacious personalized targeted therapies that overcome drug resistance and reduce side effects and toxicity.

To this end, a fundamental template for drug discovery by identifying additional druggable targets through in-depth biochemical, genomic, and molecular studies and structural investigations is needed. Drug discovery with different chemical entities or modes of action is also necessary for the development of molecular targeted therapy. In addition to direct or allosteric modulation of cellular targets, strategies for indirect manipulation of cellular targets [e.g., posttranslational modification^179^ or targeted protein degradation using proteolysis-targeting chimera (PROTAC)^180^] based on biological and functional studies for cancer-specific modulation would be applicable. Furthermore, the development of small molecule inhibitors that concurrently block signaling pathways associated with cancer cell proliferation and drug resistance and design of optimized combinatorial therapeutic strategies using molecular targeted therapy, either alone or in combination with other types of anticancer therapy (e.g., chemotherapy and immune checkpoint inhibitors), would be of importance for increased efficacy, limited toxicity, and minimal drug resistance.

Because the side effects and toxicity of targeted therapy are mediated by nonspecific inhibition of the same target in normal cells^10^, strategies for cancer cell-specific targeting are also important. A relevant example is the recent development of KRAS^G12C^ inhibitors. Since the clinical failure of farnesyltransferase inhibitors, KRAS has been considered an undruggable target^181^. In a recent study utilizing the high reactivity of cysteine, compounds that covalently bind to KRAS *via* the mutated cysteine residue and allosterically inhibit GTP binding to KRAS were designed^182^; this approach can inhibit KRAS without occupying the GTP/GDP-binding pocket on the surface and achieve specificity for mutant KRAS beyond wild-type KRAS, thus avoiding the unfavorable effects caused by inhibition of wild-type KRAS^182,183^. Based on this innovative study and a better understanding of the crystal structure of mutant KRAS, several potent KRAS^G12C^ inhibitors have been developed and approved for clinical use^183,184^; agents targeting other types of mutant KRAS, such as KRAS^G12D^, have also been developed and evaluated in preclinical settings^185,186^. Studies on molecular diagnosis and discovery of predictive biomarkers are necessary to properly select eligible populations for better efficacy and reduced toxicity^183^. Several newly developed approaches, such as next-generation sequencing technology^187^, whole-genome sequencing^188^, and machine learning^189^, can be applied to this end. In fact, artificial intelligence (AI)-based strategies^190^ are expected to be extensively utilized for the design of the structure and chemical synthetic procedures, identification of potential hits, prediction of pharmacokinetic profiles, assessment of side effects and toxicity, and drug repurposing.

Finally, emerging evidence has shown the role of the host microbiome in cancer development and progression, drug responsiveness, and therapy-induced side effects^191,192^. For example, the gut microbiome promotes the function of mutant p53 toward oncogenicity^193^ and modulates responsiveness to antitumor therapy such as anti-PD-1 immunotherapy^194^. A number of investigations into the influence of the gut microbiome on chemotherapy and anticancer immunotherapy are ongoing; however, the effect of the host microbiome on molecular targeted therapy remains elusive. Further studies are necessary to investigate the role of the host microbiome in the efficacy and toxicity of molecular targeted therapy and to identify key factors to develop safer and more efficacious therapeutic strategies based on microbiome-targeted therapy.

In summary, the present paper briefly reviews the current status of molecular targeted therapy and discusses future directions, providing novel therapeutic strategies with better efficacy and safety to improve the prognosis of cancer patients.
